# Myeloproliferative Neoplasm and Myelodysplastic Syndrome-Associated Renal Disease: A Histopathological Report of Two Cases

**DOI:** 10.7759/cureus.32388

**Published:** 2022-12-10

**Authors:** Hristo Popov, Tatiana Koleva, George S Stoyanov, Peter Ghenev

**Affiliations:** 1 General and Clinical Pathology, Forensic Medicine and Deontology, Medical University of Varna, Varna, BGR; 2 Nephrology, St. Marina University Hospital, Varna, BGR; 3 General and Clinical Pathology, St. Marina University Hospital, Varna, BGR

**Keywords:** glomerulonephritis, glomerulopathy, nephrology, myelodysplastic syndrome, myeloproliferative neoplasm

## Abstract

Myeloproliferative neoplasms (MPN) are clonal disorders of hematopoietic stem cells with a proliferation of one or more myeloid lineage and mature cell overproduction, while myelodysplastic syndrome (MDS)/MPN simultaneously show aspects of MDS and MPN, leading to partially ineffective hematopoiesis with associated dysplastic changes. This spectrum of disorders includes chronic myeloid leukemia, polycythemia vera, primary myelofibrosis, and essential thrombocythemia. MDS/MPN are classically not associated with renal complications; however, an accumulating body of evidence suggests that multiple growth factors, cytokines, endothelial damage, and an activated complement system in these patients can induce glomerulopathy, as nearly a third of these patients present with advanced renal disease on diagnosis, which is unlikely to be age or hypertension-related. In this report, we present two cases of patients with MPN/MDS, a 45-year-old male with essential thrombocythemia and a 73-year-old male with polycythemia vera, both of whom developed generalized edema and were referred to our institution from their outpatient nephrologists due to accompanying proteinuria. Renal biopsy of the first patient revealed mesangiocapillary and mesangioproliferative MPN-associated glomerulopathy. In contrast, the second patient was diagnosed with MPN/MDS-associated segmental mesangial proliferative glomerulonephritis and renal vasculature drug toxicity. Both patients were started on treatment - corticosteroid as per consensus.

## Introduction

Myeloproliferative neoplasms (MPNs) encompass clonal disorders of hematopoietic stem cells characterized by the proliferation of one or more myeloid lineages with the overproduction of mature cells. This spectrum of diseases is primarily represented by chronic myeloid leukemia, polycythemia vera, primary myelofibrosis, and essential thrombocythemia [1]. Patients with myelodysplastic syndrome (MDS)/MPN simultaneously show aspects of MDS and MPNs, with partially ineffective hematopoiesis and dysplastic changes [1].

Renal disease is not a classical complication of MPN or MDS/MPN. However, recently it has emerged that at the time of diagnosis, 11-29% of patients show stage III or IV chronic kidney disease, and about 20% show a rapid decline in glomerular filtration rate, indicative of not only age-related nephrosclerosis but also MPN/MDS-induced mechanisms [2,3]. Glomerular changes associated with MPN have been reported to include glomerular intracapillary hematopoietic cells, focal segmental glomerulosclerosis, and mesangial sclerosis [4-6].

The two largest cohorts published to date show features of chronic thrombotic microangiopathy, glomerulosclerosis, and intracapillary hematopoiesis, with these findings also being reported in smaller cohorts [7-12].

## Case presentation

Case 1

A 45-year-old male presented to the nephrology clinic with shortness of breath, dry cough, and edema of the face and lower limbs. The patient was referred to the clinic by his outpatient nephrologist due to proteinuria on a dipstick test. Previous medical history included right-sided orchiepididymitis with an abscess, which did not respond to antibiotic therapy and required bilateral scrotal incisions. Five years before the current presentation, the patient gradually developed hyperglycemia and was subsequently diagnosed with type two diabetes and started oral therapy (metformin 500mg, three times daily). Eight years prior, the patient was diagnosed with essential thrombocythemia, treated with interferon-alpha 2a, regularly adjusted in an outpatient setting, and acetylsalicylic acid 500mg once daily.

Due to his concomitant conditions, a workup for renal biopsy was initiated, with laboratory tests confirming proteinuria (3.5g/l) in 24-hour urine sample and creatinine and urea levels being 164 µmol/l and 10.4 µmol/l, respectively. Virology came negative for hepatitis B and C virus. Immunological test results came back negative except for the C3 complement fraction (1,41g/l), C4 (0.36g/l), and IgA (1.26g/l).

**Figure 1:**
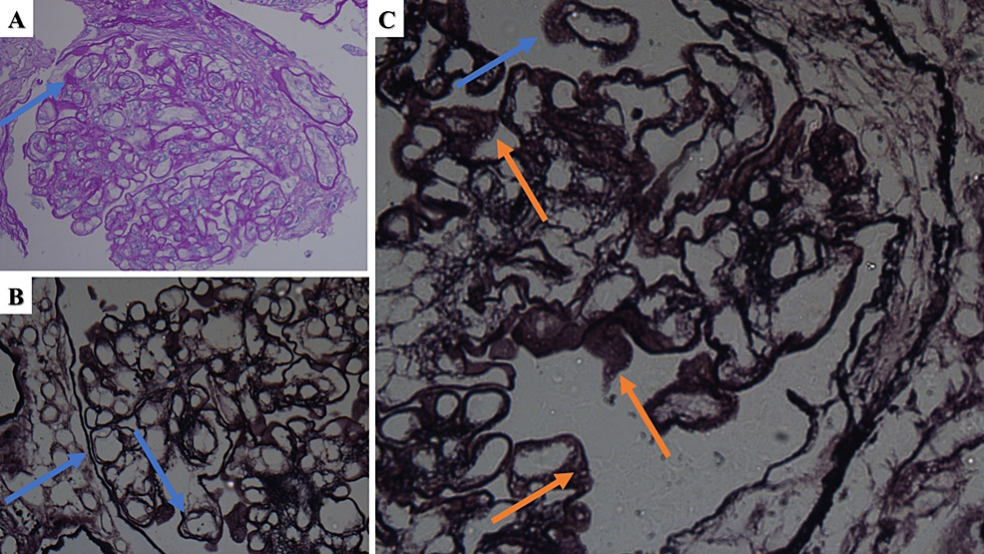
Glomerular changes in Case 1 A: endothelial intraglomerular proliferations (arrow), PAS stain, original magnification 400x; B: basement membrane tram tracking (arrows), silver impregnation (modified method), original magnification 400x; C: basement membrane spiking (blue arrow) and intramembranous vacuolation (orange arrows), silver impregnation (modified method), original magnification 1000x. PAS: periodic acid-Shiff

**Figure 2:**
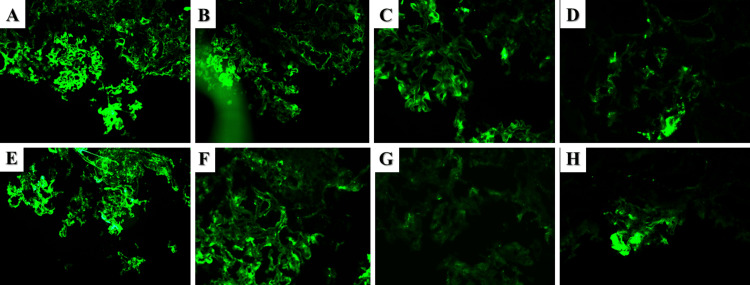
Immunofluorescent findings in Case 1 A and B: IgG, original magnification 400x; C: IgA, original magnification, 400x; D: IgM, original magnification 400x; E: C3, original magnification 400x; F: C4c, original magnification 400x; G: C1q, original magnification 400x; H: IgA, original magnification 400x

The obtained renal biopsy specimen consisted of 15 glomeruli, one completely hyalinized. The remaining glomeruli had thickened capillary basement membranes, some perforated, others with an uneven outer contour due to the formation of spikes, double-contoured capillary basement membranes, mesangial cell proliferation, and some of the glomeruli had digitation of the capillary loops (Figure 1). Immunofluorescence examination demonstrated pseudo-linear deposition of IgG (+++), C3 (+++), IgA (+++), C4c (++), and focal deposition of IgM (+++) (Figure 2). Based on his concomitant medical conditions and the morphological and immunological changes, the patient was diagnosed with mesangiocapillary and mesangioproliferative MPN-associated glomerulopathy. The patient was started on corticosteroid treatment, prednisolone pulse infusion 10mg/kg for three days, followed up by 5mg tablets orally 4-0-2, with a reduction of the bedtime dose by one tablet every 15 days and eventual reduction of the total dose to two daily tablets, as well as referred to the hematology clinic to optimize his hematological treatment, acetylsalicylic acid 500mg daily. On follow-up, cyclophosphamide pulses with 10mg/kg were attempted, but no clinical effect was noted, upon which mycophenolate mofetil was initiated with a total dose of 1g orally/daily and well tolerated with clinically beneficial effect.

Three months after the renal biopsy, he suffered abdominal trauma and underwent an emergency splenectomy due to a splenic rupture. Histopathology from the spleen showed obliteration of the splenic structure and multiple megakaryocytes arranged in clusters.

On follow-up, the patient was stable, with a steady improvement in renal function: 24-hour proteinuria 1.28 g/l, serum creatinine, and urea of 118 mcmol/l and 9.4mmol/l as per the latest tested values. Sadly three years after the initial diagnosis, the patient expired from coronavirus infection.

Case 2

**Figure 3:**
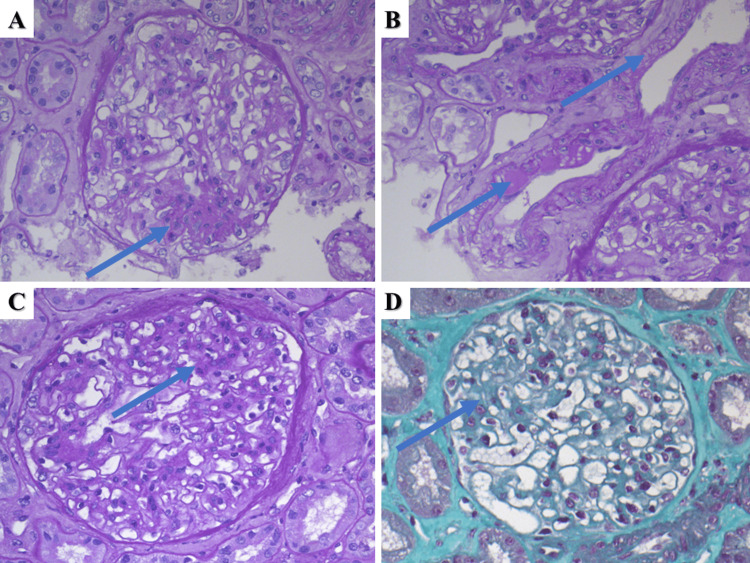
Histopathological changes in Case 2 A: mesangial cell proliferation (arrow), PAS stain, original magnification 400x; B: drug toxicity associated vascular changes (arrows), PAS stain, original magnification 400x; C and D: mesangial fibrosis, PAS and Mason trichrome stains, original magnifications 400x. PAS: periodic acid-Shiff

**Figure 4:**
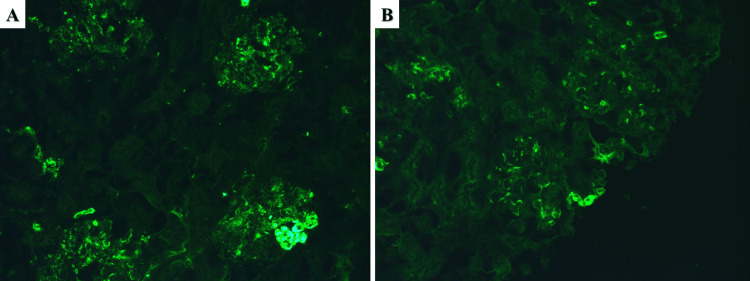
Immunofluorescent findings in Case 2 A: IgM, original magnification 200x; B: C1q, original magnification 200x

A 73-year-old male was referred to our nephrology clinic due to lower limb and periorbital edema for the previous three months without any change in the volume or color of the urine. Outpatient nephrology-prescribed laboratory tests revealed hypoproteinemia at 48.3 g/l, serum albumin at 24 g/l, and a significant amount of protein in the urine of 3.18 g/l in the 24-hour sample. Previous medical history included hypertension diagnosed 10 years prior and polycythemia vera diagnosed 13 years prior and treated with ruxolitinib for the past several years, both of which are under adequate medication control with regular outpatient follow-ups. Hospital laboratory tests returned negative for virology (hepatitis B and C) and non-elevated for circulating antibodies; serum creatinine and urea were normal, 78 mcmol/l and 7.5mmol/l, respectively. Due to the symptoms of the patient and the negative laboratory findings, a renal biopsy was obtained with histopathology of the specimens reporting a total of 47 glomeruli, three of which were completely hyalinized. The remaining glomeruli had moderately thickened capillary basement membranes and an expanded eosinophilic mesangium with fibrotic changes (Figure 3). More than half of the glomeruli had moderate mesangial cell proliferation, with four to five cells per mesangial axis (Figure 3A). The tubular system had minimal changes consisting of few protein casts and fibrosis (less than 25%); the interstitium had minimal changes consisting of lymphocyte infiltration and fibrosis in less than 25% of the obtained specimen. Arteries revealed subintimal fibrosis and myoelastofibrosis with medication-associated vascular toxicity (Figure 3B). Immunofluorescence showed mesangial deposits of IgM (++) and focal C1q fraction of the complement (+) (Figure 4). MPN/MDS-associated segmental mesangial proliferative glomerulonephritis was diagnosed based on the morphology, immunological deposits, and patient medical history. The patient was started on corticosteroid therapy - prednisolone pulse infusion 10mg/kg for three days, followed by 5mg tablets orally 4-0-2, with a reduction of the bedtime dose by one tablet every 15 days and referred to a hematologist to reevaluate the myeloproliferative disorder. On follow-up, mycophenolate mofetil was initiated with a total dose of 200mg orally/daily and well tolerated with clinically beneficial effects.

On follow-up, the patient is stable with an adjustment of the treatment for the hematological condition with the addition of acetylsalicylic acid 500mg daily, with a steady improvement of renal function: serum protein levels 74.8 g/l, serum albumin 48 g/l, creatinine 78 mcmol/l, urea 8.2 mmol/l, as per latest tested values.

## Discussion

In renal pathology in patients with MDS/MPN, segmental and global glomerulosclerosis, mesangial sclerosis, and hypercellularity are the leading chronic changes reported in case series [5,7,13]. Age, gender or hypertensive disease-related changes in the kidney are suggested to be unlikely to be related to chronic glomerular changes in MPN and MDS/MPN patients. It is more likely that the MPN causes the changes; hence, MDS/MPN can be viewed as a cause for chronic glomerulopathies. It is also suggested that prolonged damage to the podocytes and endothelium are likely important mechanisms responsible for glomerular lesions. Multiple cytokines and growth factors (e.g., interferon-γ, interleukin-6, platelet-derived growth factor, transforming growth factor-β, and fibroblast growth factor) documented in patients with MPNs and other hematological diseases can directly cause endothelial damage [13-19].

In addition, growth factors may directly contribute to mesangial cell proliferation and mesangial and subendothelial matrix production in a manner similar to the development of myelofibrosis in some MPN and MDS/MPN patients [19-24].

Marginated platelets and cells of extramedullary hematopoiesis can locally produce growth factors and, together with hyperviscosity, increase endothelial damage [19]. In the first patient, however, no megakaryocytes were demonstrated in the lumens of glomerular capillary loops on CD61 staining. But abundant deposition of IgG, IgM, IgA, C3, and C4c was observed, with a negative reaction for C1q, Kappa, and Lambda, which may be associated with the activation of neoplastic cells, which produce immunoglobulins and activate the complement system.

It also cannot be ruled out that current therapies with tyrosine kinase inhibitors contribute to chronic endothelial damage in MPN and MDS/MPN patients. JAK2 (Janus kinase 2) inhibitors may have similar effects [9,25]. Furthermore, the second of our cases showed adverse vascular effects in the renal intraparenchymal arteries, nodular hyaline sclerosis of arterioles, which may be attributed to his ruxolitinib treatment, which would further have an adverse effect on renal function.

In other reports, it is discussed that it can be associated with MPN-associated or MDS/MPN-associated renal disease and glomerulonephritis with C3 deposition, reminiscent of infection-associated glomerulonephritis [19]. This observation could be interpreted as an activation of the complement system due to soluble factors secreted by the neoplastic cells or a subtype of C3 glomerulopathy developing in the context of a myeloid disease.

## Conclusions

Chronic kidney disease associated with MPN and MDS/MPN is characterized by proteinuria, hematuria, and chronic renal failure. The histological picture is diverse in patients with MPN/MDS, with other authors reporting different findings, the most common of which are mesangial sclerosis and hypercellularity, segmental and global glomerulosclerosis. In our case, the histological changes are mainly represented by double-contoured capillary basement membranes, spiking of the capillary basement membranes in the type of membranous nephropathy, digitations and lobularization of the capillary loops in the style of mesangiocapillary glomerulonephritis, mesangial cell proliferation, and segmental glomerulosclerosis. These patients should be monitored for early detection of low-grade proteinuria and microscopic hematuria, risk factors, and late-onset renal involvement of the chronic glomerulonephritis type.
